# How a disruption of the competition between HIF-1 and p53 for limiting p300/CBP by latent viruses can cause disease

**DOI:** 10.18632/genesandcancer.178

**Published:** 2018-05

**Authors:** Hanan Polansky, Hava Schwab

**Affiliations:** The Center for the Biology of Chronic Disease (CBCD), Valley Cottage, NY, USA

CBP and p300 are considered the most heavily connected coactivators in the mammalian protein-protein interaction network [1] with at least 315 different cellular and viral interacting partners [2]. CBP and p300 are histone acetyltransferases that control the transcription of numerous genes in humans, viruses, and other species.

CBP/p300 is a 300 kDa protein that has a CH2 domain, which contains its acetyltransferase activity, and five binding domains [3]. Although two separate genes encode CBP and p300, they share a 61% sequence identity, and are often mentioned together as CBP/p300 [3]. Many studies showed that competition for the limiting CBP/p300 is an important mechanism used by the cell to regulate transcription and cellular behavior. This commentary discusses two of these studies [4] [5] and connects the observations reported in these studies to the Microcompetition Model.

HIF-1α, a subunit of the hypoxia-inducible factor-1 (HIF-1) transcription factor, is regulated in an oxygen-dependent manner. Under normal oxygen conditions it is inactive and made at low levels. Under hypoxic conditions, it is stabilized and activated. The tumor suppressor p53 is another protein that is active under hypoxic conditions.

Using differential equations and a dimensionless state variable, Zhou et al. [4] determined the effect of p300 on the steady-state concentrations of proteins. They discovered that under hypoxic conditions HIF-1α and tumor suppressor p53 compete for binding to the co-activator p300. They showed that the co-activator p300 is required for full transcriptional activity of both p53 and HIF-1. According to Zhou et al., this competition indicates that p300 is limiting.

To investigate the cross-talk between HIF-1α and p53, Ruas et al. [5] performed ChIP analyses to examine the recruitment of CBP to HIF-1α and p53 target gene promoters under hypoxic conditions. The results of the ChIP analyses showed that under this condition the levels of CBP on target gene promoters are reduced compared to the maximum binding levels. Based on these results, Ruas et al. concluded that CBP/p300 is limiting, and that HIF-1α and p53 compete for recruitment of the limiting amounts of CBP/p300 to their target gene promoters, and that this competition affects the transcription of these genes.

These studies showed that competition between the cellular transcription factors HIF-1α and p53 for binding the limiting p300 is an important regulator of transcription. According to the Microcompetition Model, a disruption of this regulation can cause many diseases. The Microcompetition Model was first described in the book ‘Microcompetition with Foreign DNA and the Origin of Chronic Disease.’ [6][7] The model centers on one type of disruption of this regulation caused by viruses that include the strong cis-regulatory element found their promoters/enhancers called the N-box. This element binds the cellular p300·GABP transcription complex during the latent phase. Some common viruses that include an N-box are the Cytomegalovirus (CMV), Epstein-Barr virus (EBV), Herpes Simplex Virus 1 (HSV-1), Human T-cell lymphotropic virus (HTLV), and Human Immunodeficiency Virus (HIV). Since p300 is limiting, the p300·GABP complex is limiting. Therefore, the viral N-boxes decrease the availability of p300·GABP in the cell. The result is abnormal expression of the cellular genes that also bind the transcription complex. Those genes that are transactivated by the p300·GABP complex synthesize fewer proteins, while those that are transrepressed by the complex synthesize more proteins. The abnormal levels of these cellular proteins can cause diseases, including cancer, diabetes, atherosclerosis, and obesity. In the book, there is a list of many human genes that bind the p300·GABP complex, and evidence that shows that these genes express abnormal levels of their proteins in these diseases, as suggested by the model.
